# Toremifene-induced bilateral macular crystalline deposits with cystic cavitation: a case report

**DOI:** 10.3389/fmed.2026.1840738

**Published:** 2026-07-23

**Authors:** Chunhuan Niu, Fei Wang, Jiao Li, Xiaotong Sun, Xiaofeng Xie, Yane Gao

**Affiliations:** 1 Affiliated Eye Hospital of Shandong University of Traditional Chinese Medicine, Jinan, Shandong, China; 2 Shandong Provincial Key Laboratory of Integrated Traditional Chinese and Western Medicine for Prevention and Therapy of Ocular Diseases, Jinan, Shandong, China

**Keywords:** drug-induced retinopathy, macular crystalline deposits, macular cystic vacuolization, selective estrogen receptor modulators (SERMs), toremifene

## Abstract

Tamoxifen-induced retinopathy is well recognized and frequently reported in clinical practice. By contrast, ocular adverse events associated with the structurally similar agent toremifene remain rarely described. We present a case of bilateral visual impairment in a patient receiving long-term oral toremifene citrate for secondary prevention after breast cancer surgery. Ophthalmic evaluation showed yellowish-white crystalline deposits within the macular retinal layers in both eyes. Optical coherence tomography (OCT) revealed cystic vacuolization in the bilateral foveal regions, with features closely resembling tamoxifen-induced retinopathy. A clinical diagnosis of toremifene-associated retinopathy was established. This case may provide reference for clinicians when judging the appropriate timing of toremifene withdrawal. Should drug cessation be considered, a joint assessment by ophthalmologists and oncologists should be performed to balance the risks of tumor progression and ocular damage before reaching a clinical decision. In addition, this case supplements available evidence regarding toremifene-related ocular toxicity and raises clinical awareness of this condition.

## Introduction

Retinal crystalloid deposits may occur in various systemic disorders and are also linked to several medications, including the anticancer agent tamoxifen, the anesthetic methoxyflurane, and the oral tanning agent canthaxanthin (1, 2). Among these, tamoxifen-associated retinopathy is a well-documented adverse reaction, characterized by bilateral intraretinal crystalline deposits in the macular region, with or without accompanying cystic changes (1, 3, 4).

Like tamoxifen, toremifene is a non-steroidal anti-estrogen and a selective estrogen receptor modulator (SERM). Both agents are widely used for secondary prevention in hormone receptor-positive breast cancer to reduce recurrence risk in high-risk patients. The two agents share highly homologous molecular structures and analogous pharmacological mechanisms. To date, there have been no definitive clinical reports documenting ocular adverse effects associated with toremifene administered at the standard 60 mg daily dose. We report a female patient who developed bilateral macular crystalline deposits with cystic vacuolization after five consecutive years of postoperative toremifene treatment. Based on clinical findings and literature review, we discuss the clinical features, pathogenesis, diagnosis, and management of toremifene-associated retinopathy, to support the safe clinical use of this drug and the recognition of its ocular adverse events.

## Case description

A 38-year-old female presented to the ophthalmology clinic in October 2025 with progressive bilateral visual deterioration over the previous year, which had worsened in the last 3 months. She was diagnosed with breast cancer 5 years earlier and underwent right mastectomy. Postoperatively, she received subcutaneous injections of the gonadotropin-releasing hormone (GnRH) agonist goserelin as adjuvant therapy (discontinued 2 years before presentation) and continuous oral toremifene citrate 20 mg daily for 5 years, with a cumulative dose of 36.5 g. She had undergone bilateral total thyroidectomy for thyroid cancer 7 years prior and had been on long-term levothyroxine sodium for hormone replacement. She had no history of diabetes, hypertension, primary ocular disease, or intraocular surgery, and denied other systemic disorders associated with macular damage.

**FIGURE 1:**
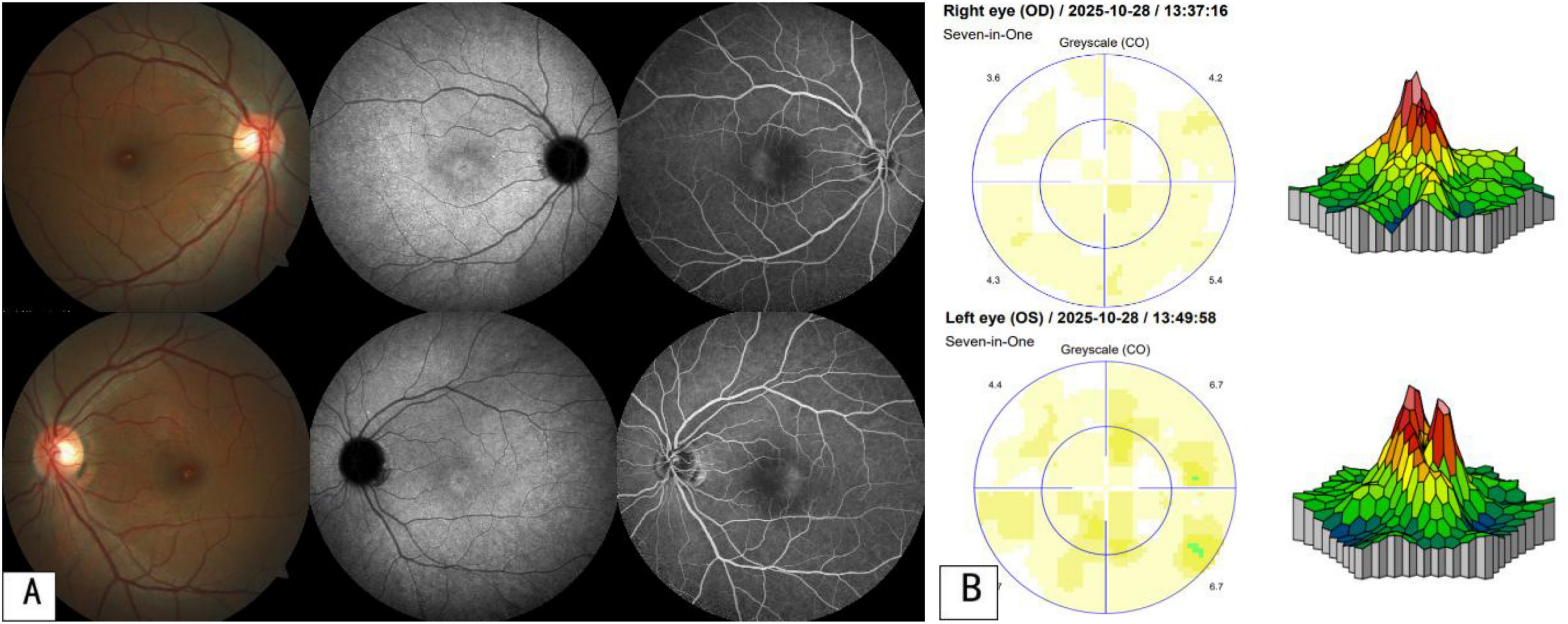
**(A)** Fundus photography shows scattered yellowish-white crystalline deposits in the macular region of both eyes. Late-phase ICGA demonstrates mild fluorescein leakage in the perifoveal areas bilaterally; FFA shows mild fluorescein leakage in the temporal perifoveal region bilaterally. **(B)** A 10° visual field test revealed patchy decreases in light sensitivity in the central visual fields of both eyes; mERG reveals decreased response amplitudes in the central, paracentral, and pericentral regions.

**FIGURE 2:**
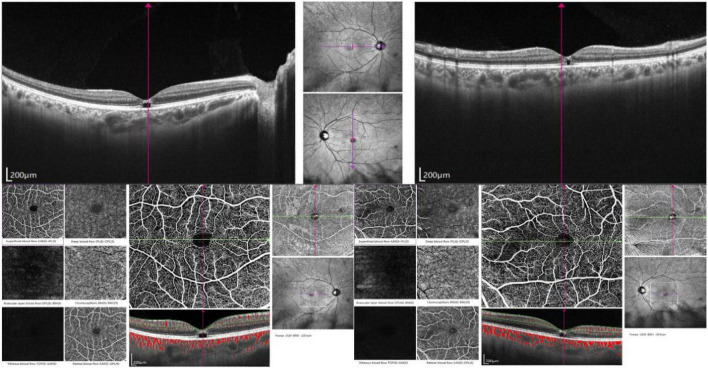
Optical coherence tomography angiography (OCTA) shows cystic vacuolization in the outer retinal layers at the fovea in both eyes, with complete loss of the ellipsoid zone, while inner retinal layers remain intact. Retinal vasodilation and reduced macular blood flow density are observed in the bilateral macular regions. OCTA parameters: Superficial blood flow ILM(0)-IPL(9), Deep blood flow IPL(6)-OPL(9), Avascular layer blood flow OPL(6)-BM(0), Choriocapillaris BM(0)-BM(29), Vitreous blood flow TOP(0)-ILM(0), Retinal blood flow ILM(0)-OPL(6).

On examination, the best-corrected visual acuity (BCVA) was 20/40 in the right eye and 20/33 in the left eye, with normal intraocular pressures (IOP) bilaterally. Anterior segment examination was unremarkable. Scattered yellowish-white crystalline deposits were observed in the macular retinal layers. Late-phase indocyanine green angiography (ICGA) demonstrated mild fluorescein leakage in the perifoveal areas bilaterally; fundus fluorescein angiography (FFA) showed mild fluorescein leakage in the temporal perifoveal region bilaterally (Figure 1A). A 10° visual field test revealed patchy decreases in light sensitivity in the central visual fields of both eyes; multifocal electroretinography (mERG) showed decreased response amplitudes in the central, paracentral, and pericentral areas (Figure 1B). On optical coherence tomography angiography (OCTA), cystic vacuolization was identified in the outer retinal layers at the fovea in both eyes, with complete loss of the ellipsoid zone and preserved inner retinal layers. Retinal vasodilation and reduced macular blood flow density were also noted bilaterally (Figure 2).

A clinical diagnosis of toremifene-associated retinopathy was established based on the patient’s long-term medication history, typical multimodal imaging findings, and exclusion of other causes of maculopathy. Given the risk of disease progression, the decision to discontinue toremifene required multidisciplinary assessment with an oncologist to balance the risks of tumor recurrence and worsening ocular damage. The patient was referred to oncology for further evaluation. After comprehensive oncologic review and in view of her prolonged disease-free survival, toremifene was discontinued following informed consent. On telephone follow-up, the patient reported no significant change in subjective visual symptoms after cessation. Regular ophthalmic follow-up and close monitoring of her general condition were recommended.

## Discussion

Toremifene is a chlorinated derivative of tamoxifen; both are selective estrogen receptor modulators (SERMs) with comparable clinical efficacy, widely utilized in the treatment of early- and advanced-stage hormone receptor-positive breast cancer, as well as in secondary prevention (5, 6). No explicit documentation of adverse reactions related to retinal toxicity or retinopathy is found in its Summary of Product Characteristics (SmPC). Only 4 spontaneous reports of toremifene-induced retinal toxicity were retrieved from the FDA Adverse Event Reporting System (FAERS) database. This low reporting volume may be attributable to underreporting of clinical cases, a lack of routine ophthalmological screening in standard clinical practice, and the insidious onset and slow progression of this type of retinopathy. Previous studies have indicated that replacement of the hydrogen atom in the ethyl side chain of toremifene with a chlorine atom alters its metabolic pathway, thereby resulting in a significantly lower incidence of adverse reactions (e.g., ocular toxicity, vascular embolism) compared with tamoxifen (6). However, these conclusions were largely derived from early studies with small sample sizes and short follow-up durations. With the widespread clinical application of toremifene and the extension of follow-up periods, its potential ocular adverse reactions have been gradually identified. Some studies have identified no statistically significant disparity in the prevalence of retinal lesions among patients with breast cancer receiving 3 years of tamoxifen versus toremifene (7). It indicates that the risk of ocular toxicity linked to both agents may follow a time-dependent pattern, underscoring the need for sustained ophthalmic monitoring in patients on extended treatment to guard against the onset of retinal pathology.

Selective estrogen receptor modulator-associated retinopathy is characterized by crystalline deposits, which are predominantly fine, highly reflective, yellowish-white crystalline bodies distributed primarily in the retinal nerve fiber layer and inner plexiform layer, particularly in the macular region. In the early stages, these deposits present as scattered dots, which may gradually coalesce with disease progression. Currently, it is widely accepted that the pathogenesis of this condition is not attributed to the drug’s modulatory effects on estrogen receptors, but rather mediated by the drug’s cationic amphiphilic properties, analogous to the pathogenesis of chloroquine-associated retinopathy (8, 9). Toremifene possesses both hydrophilic and lipophilic properties, enabling it to readily cross biological membrane barriers, accumulate in corneal and retinal tissues, and form stable complexes with cellular lipids. When local drug concentrations exceed the solubility limit, toremifene binds to polar lipids to form crystalline deposits, which accumulate in lysosomes and induce oxidative cellular damage (10–13). These deposits not only cause mechanical damage to retinal tissue but also interfere with normal local physiological functions. As the macula serves as the core region for central vision, structural damage to this area directly leads to symptoms such as decreased visual acuity and visual distortion in patients (14). Histopathological studies have confirmed that these crystal-like deposits may essentially represent axonal degeneration in the nerve fiber layer surrounding the fovea (15).

Estrogen receptors α and β are expressed in the retina, retinal pigment epithelium, and choroid of the eye (16). Activation of these receptors plays a crucial role in maintaining the normal structure and function of ocular tissues, regulating retinal neuronal survival, inhibiting oxidative stress-induced damage, and preserving the stability of the blood-retinal barrier (12, 17, 18). Through these receptors, estrogen can downregulate pro-apoptotic proteins and upregulate anti-apoptotic proteins, thereby reducing apoptosis of retinal pigment epithelium cells and maintaining the stability of the retinal microenvironment (19, 20). Meanwhile, estrogen acts on Müller cells and retinal ganglion cells to alleviate oxidative stress, inhibit apoptosis, and promote axonal regeneration (21). As an estrogen receptor antagonist, toremifene competitively binds to estrogen receptors, antagonizing the protective effects of estrogen on the eye. This leads to disruption of the blood-retinal barrier and accumulation of interstitial fluid, thereby inducing macular cystoid degeneration (18). The incidence of macular cystoid degeneration is higher in postmenopausal women (22, 23), which is associated with a sharp decline in estrogen levels and the subsequent loss of its protective effects after menopause; the administration of anti-estrogen drugs further exacerbates this pathological process (17, 22, 24). Previous investigations have demonstrated an association between tamoxifen and macular holes as well as their precursor lesions. Females administered tamoxifen exhibit a substantially higher risk of developing macular holes compared with age-matched controls (4.12% versus 0.82%, *P* = 0.0001) (25). Such pathological changes are plausibly mediated by neurodegeneration of Müller glial cells (26). Although this patient had not reached natural menopause, she received goserelin therapy for 3 years after breast cancer surgery, which induced artificial amenorrhea and persistent hypoestrogenism. Goserelin had been discontinued 2 years prior to the onset of ocular symptoms; nevertheless, the long-term hypoestrogenic state induced by the drug may exert sustained long-term impacts on retinal tissue. On this basis, the persistent anti-estrogenic effect of toremifene further adds to this condition, and the two factors may exert synergistic impacts. Accordingly, the development and progression of macular lesions in this patient are likely attributable to the combined effects of toremifene and goserelin, rather than toremifene monotherapy alone.

In addition to its estrogen-antagonistic effects, the occurrence of toremifene-associated macular cystic vacuolization may also be related to the drug’s direct toxicity to Müller cells and its tissue accumulation effects. Müller cells are important glial cells in the retina that are involved in maintaining the stability of the retinal microenvironment, nutrient metabolism, and signal transduction; their functional abnormalities or damage directly compromise the structural integrity of the retina (27). Studies have identified similarities between tamoxifen-related retinopathy and the pseudocystic foveal cavitation characteristic of type 2 idiopathic macular telangiectasia (MacTel2). Both conditions feature crystalline deposits and cystic spaces, indicating that Müller cell dysfunction may constitute a central pathogenetic link (1, 28). In this case, fluorescein fundus angiography combined with indocyanine green angiography (FFA + ICGA) revealed only mild fluorescein leakage, indicating that the blood-retinal barrier had not been severely impaired. It is speculated that toremifene may affect the ion channel function or metabolic processes of Müller cells, leading to intracellular fluid retention, cell swelling, and subsequent intraretinal cystic cavities (29, 30). Furthermore, toremifene is rapidly absorbed after oral administration, with a mean half-life of 11 days (range, 4–20 days); regular daily dosing can easily lead to slow accumulation of the drug in the body. In this case, the patient had accumulated a total dose of 36.5 g over a 5-year treatment period, which far exceeded the observation periods of early studies. The accumulated concentration of the drug in retinal tissue may have exceeded the tolerance threshold, which is a key reason why its ocular toxicity was not fully identified in earlier studies.

After discontinuation of toremifene, damage to Müller cells was partially reversed, and cellular function gradually recovered. This may explain why some patients experienced improved vision and partial resorption of crystalline deposits following discontinuation; however, recovery from macular cystic vacuolization was relatively slow, possibly due to irreversible damage to the inner retinal layers. Currently, there is no unified standard for the treatment of toremifene-associated retinopathy, and the extent of lesion reversal after discontinuation, as well as long-term prognosis, still require confirmation through large-scale, long-term follow-up studies.

This case provides important insights for clinical practice: ① Emphasize long-term ophthalmic screening: Although the incidence of toremifene-induced ocular toxicity is low, patients receiving long-term therapy should still undergo regular eye examinations. For high-risk patients who have been on the medication for more than 3 years or who have low estrogen levels due to combined endocrine therapy, it is recommended to perform fundus examinations and optical coherence tomography (OCT) scans every 6–12 months to detect signs of retinal pathology at an early stage. ② Promote multidisciplinary collaborative care: The core of managing toremifene-associated retinal pathology lies in balancing the risks of tumor recurrence and progression of ocular lesions; this requires joint assessment by ophthalmologists and oncologists to develop individualized treatment plans. For patients with a prolonged disease-free survival and low risk of tumor recurrence, treatment discontinuation can be considered following informed consent alongside close follow-up. For those at high risk of disease recurrence, endocrine therapy can be maintained under rigorous ophthalmic surveillance, or switched to alternative endocrine agents with superior ocular safety profiles. Raloxifene exhibits comparable efficacy to tamoxifen in breast cancer prevention with a lower incidence of systemic adverse reactions. More importantly, unlike the potential retinal toxicity associated with tamoxifen, raloxifene exerts protective effects on retinal structure and function, with particular beneficial potential for photoreceptor cells (31, 32).

This study is a single-case clinical report and has some limitations. First, the patient had a prior history of goserelin administration. Long-term goserelin use induces estrogen deficiency, which may subsequently damage macular architecture. Although goserelin had been discontinued 2 years before the onset of the patient’s ocular symptoms, and the patient presented with yellow-white crystalline precipitates in addition to macular structural damage, we cannot entirely rule out a potential synergistic interaction between goserelin and toremifene that jointly impairs the retina. Second, the scheduled ophthalmic follow-up reassessments could not be completed due to the patient’s stable subjective ocular symptoms and personal matters. The absence of follow-up imaging precluded definitive evaluation of whether retinal structural damage remained stable, progressed, or partially reversed after drug discontinuation; this also reduces the strength of the causal inference regarding the retinal toxicity induced by toremifene. Third, serum hormone profiles, relevant immunological assays and molecular biological examinations were not performed in this case, making it difficult to further explore the specific pathogenic mechanisms underlying toremifene-mediated retinal toxicity. Finally, conclusions drawn from this single patient are limited by the small sample size and lack generalisability across broader populations.

## Conclusion

Long-term oral use of toremifene can induce bilateral macular crystalline deposits and cystic cavities, and its ocular toxicity may present in a time-and dose-dependent manner. In clinical practice, breast cancer patients on long-term toremifene therapy should undergo regular ocular screening to enable early detection of lesions; once relevant retinal lesions appear, multidisciplinary collaboration between ophthalmology and oncology is required. This case provides practical evidence to deepen clinical understanding of toremifene-associated retinal lesions; however, the exact causal relationship and pathogenesis still require further validation through large-scale, prospective clinical studies.

## Data Availability

The original contributions presented in this study are included in the article/supplementary material, further inquiries can be directed to the corresponding authors.
